# Lectures on the Theory and Practice of Midwifery, Delivered in the Theatre of St. George’s Hospital

**Published:** 1844-10

**Authors:** 


### REVIEWS.

---

Art. III.—*Lectures on the Theory and Practice of Midwifery. Delivered in the Theatre of St. George's Hospital.* By Robert Lee, M.D, F.R.S , &c. Illustrated with numerous wood engravings. Philadelphia: Ed. Barrington & Geo. D. Haswell. 1844. 8vo. pp. 540.

*A Practical Treatise on Midwifery: exhibiting the present advanced state of the Science.* By F. J. Moreau, Officer of the Legion of Honor, and of the Order of Leopold; Professor of Midwifery and the Diseases of Women and Children in the Faculty of Medicine of Paris, &c. Translated from the French by Thomas Forrest Betton, M.D., and edited by Paul B. Goddard, A.M., M.D., &c. *With* 80 *plates, comprising numerous separate illustrations.* Philadelphia: Cary & Hart, for G. N. Loomis. 1844. 4to. pp. 235.

We have already expressed the favorable opinion which we entertain of the first of these works, and now return to it for the purpose of presenting a few extracts from it, which, unless we are very much deceived, will justify our judgment. The subject is one in which our readers necessarily take the deepest interst, and although we may not be able to furnish them with anything from the pages of Dr. Lee's Lectures decidedly novel, we are quite sure that every passage will indicate in the author a discriminating, practical mind, enlightened by learning, and extensive observation. If we have studied these lectures to any advantage, the quality for which they are most remarkable is their elaborateness. There is a fullness in the information which they embrace on all the points discussed, not always found in works of greater size. The details, without being unnecessarily tedious, are so ample, that the curiosity of the reader is fully satisfied, and he lays down the book with the feeling that he has been instructed in the important business to which it is devoted. As a specimen of this excellence, we quote the following from a multitude of similar passages. Enlarging, in his introduction, on the dangers attendant upon child-birth, the author remarks:

"That such occurrences are not uncommon, the reports of public institutions in different countries afford too abundant proof. 219,333 women were delivered in the kingdom of Wurtemberg from the year 1821 to 1825, of whom there died 1248, or one in 175. At Hamburg, 5.57 women died in 1000 who have been pregnant or delivered. Of 448,356 females who died in the Prussian States, in the course of fifteen years, between the ages of fourteen and forty-five, 70,-215, or nearly one-sixth died either immediately in the act of delivery or in child-bed; and of 108 of the infants born, 1 in 108 cost the mother her life. In the mortality table for the city of New York for a period of thirty-two years, viz: from 1805 to 1836, both inclusive, it is stated that the births during this period ranged from 200,000 to 300,000; and that the whole number of deaths from child bed were only 1038. In the Dublin Lying-in Hospital, from the 8th day of December, 1837, to the 31st day of December, 1842, there were 132,979 women delivered, of whom 1488 died. Of 16,654 women delivered in the same institution during a period of seven years, commencing November 1826, there were 1956 difficult cases, and of these 164 proved fatal. There were 409 preternatural presentations; 24 cases of uterine hemorrhage before the birth of the child, and 64 between the expulsion of the child and placenta; 66 of retention of the placenta; 30 of puerperal convulsions; 240 of twins, and 4 of triplets; 97 of prolapsus of the funis; and 88 of puerperal fever, 56 of which were fatal. In the British Lying-in Hospital, Brownlow street, 33,627 women were delivered between the years 1749 and 1824, of whom 433 died: in the first ten years 1 in 42; in the second ten years 1 in 50; in the third ten years 1 in 53; in the fourth ten years 1 in 60; in the fifth ten years 1 in 288; in the sixth ten years 1 in 231; in the 7th ten years 1 woman in 274.

"During the last fifteen years, in public institutions and in private practice, I have seen 55 cases of difficult and protracted labour, in which recourse was had to the forceps; above 100 in which delivery was effected by the operation of of craniotomy, distortion of the pelvis being the most common cause of the difficulty; 35 in which premature labour was induced; 62 cases of arm presentation; 36 in which the placenta was adherent to the neck of the uterus, and 38 in which it was detached from the fundus and produced dangerous hemorrhage; 7 fatal cases of retention of the placenta, and 19 in which great difficulty and danger were produced by portions of the placenta, or the entire mass, being left within the uterus beyond the usual period; 46 labours complicated with puerperal convulsions, and upwards of 200 of puerperal fever, or inflammation of the uterine organs in the puerperal state. Written histories having been preserved of these cases, they have now been collected and arranged for publication, in the hope that they may be found to illustrate, confirm, or correct, the rules laid down by systematic writers for the treatment of difficult labours, and supply that course of clinical instruction in midwifery, the want of which has been so often experienced by practitioners at the commencement of their career."

The following paragraph is of the same character, and contains valuable statistics:

"It has been observed by all practitioners, that, in a very great proportion of cases, it is in the first pregnancy or labour that puerperal convulsions occur. 'Women are far more liable,' says Dr. Denman, 'to convulsions in first than in subsequent labours; and then, it is said, more frequently when the child is dead than when it is living. But when women have convulsions the death of the child ought generally to be esteemed rather an effect than a cause, as they have often been delivered of living children when they were in convulsions, or of dead, or even putrid children, without any tendency to convulsions. Some women have also had convulsions in several successive labours: but having had them in one, they generally, by the precautions taken, or some natural change, escape them in future. Lastly, I was for many years persuaded that convulsions only happened when the head presented; but experience has proved that they sometimes occur in preternatural presentations of the child.' Of 19 cases recorded by Dr. Joseph Clark, 16 were first children. Of 48 related by Dr. Merriman, there were 36 instances in which it was the patient's first labaur. Of 30 cases which occurred to Dr. Collins, 29 were in women with their first children; and the other single case was a second pregnancy, but in a woman who had suffered a similar attack with her first pregnancy. 14 of the 32 children (two of the women having had twins) were born alive. In 18 of the 30 the convulsions subsided after delivery; in 10 the fits occurred both before and after; and in 2 the attack did not come on till after delivery. In 15 of the 30 the patients were delivered by the natural efforts; in 6 delivery was effected by the forceps; in 8 by the perforator and crotchet; and in one the feet presented. Two of the children were born putrid. Five of the women died. In 6 of the 48 cases related by Dr. Merriman the convulsions did not occur till after delivery. Five of these patients recovered; the other, after the epileptic attack, became maniacal, but appeared to be gradually recovering when, at the end of three weeks from the first seizure, she was attacked with another fit, and died. All the children were alive. In 3 cases the women were pregnant of twins. In 2 of these cases the attack of convulsions occurred in the interval between the births of the two children. All the women were delivered without artificial assistance; 2 of them recovered; and 3 of the children were born alive. In 11 cases the delivery was effected by the forceps. All these women recovered, and 3 of the children were born alive. In 9 cases the perforator was employed. Seven of the women recovered. In 4 cases the operation of turning was resorted to; 2 of the women recovered; all the children were dead born. In one case the woman died undelivered. In 14 cases the children were born without extraordinary assistance. 10 of these women recovered, and 5 of the children were born alive. Thus 37 women recovered, and 11 died. 17 children were born alive (including the 6 born before the mothers were attacked with convulsions); 34 were born dead. Dr. Ramsbotham has related the histories of 26 cases; of which 10 proved fatal. 13 occurred before delivery, 10 during labour, and 4 after. Dr. Ingleby relates 35 cases; of which, 11 were fatal. Mauriceau 42; 7 during pregnancy, 3 of which were fatal; 19 during labour, 11 of which ended fatally; and 16 after delivery, of which 5 were fatal"

The reader will perceive from these quotations that this volume possesses great value as a work of reference. It is a store-house of facts from which the student may enrich his mind, and to which the practitioner may apply to refresh his memory. How laboriously the author has been engaged in the investigation of the various topics discussed in his work, and with what clearness and decision he expresses his conclusions, may be gathered from the following extract, taken from the lecture on the *uterine inflammation of puerperal women:*

We know from the ancient writers that puerperal women have been liable in all ages to attacks of inflammation of the uterine system. In the works, however, of the earlier authors, its history is short and imperfect; and it is probable that the disease did not attract the particular attention of the physicians before the middle of the 17th century, when it occurred at Paris as a malignant epidemic in the lying-in wards of the Hôtel-Dieu, and the bodies ot those who died with it were found to be full of abscesses. Since that period it has often been observed in the principal cities and lying-in hospitals of Europe, both in a sporadic and epidemic form, and has been denominated by medical authors puerperal fever, child-bed fever, puerperal peritonitis, peritoneal fever, puerperal typhus, the epidemic disease of lying-in women. Upwards of 200 cases of this disease have come under my observation since the spring of 1827, the histories of 160 of which have been reduced into a tabular form. I have watched the symptoms and progress of these cases with the closest attention; observed the effects of the different remedies employed, and where death took place I carefully examined the alterations of srtucture in the uterine and other organs. The bodies of a great number of the women have been inspected by myself personally: this task was not delegated to others ignorant of the pathology of the uterine system; and in all, without one single exception, there was some morbid change, decidedly the effect of inflammation, either in the peritoneal coat of the uterus or of its appendages, in the muscular tissue, the lining membrane, or in the veins or absorbents of the organs, which accounted in a most complete and satisfactory manner for all the constitutional disturbance which had taken place during life. The ganglia and nerves of the uterus were the only structures which were not examined. These observations entirely subvert the opinion that there is a specific, essential, or idiopathic fever, which attacks puerperal women, and which occurs independently of any local affection in the uterine organs, and may even prove fatal without leaving any perceptible change in the organization of any of their different textures. As the constitutional symptoms thus appear invariably to derive their origin from a local cause, as the local affection is always uniform, and nothing else is so in the history of the disease, I have thought that it would be more philosophical, and more consistent with the correct principles of nosological arrangement, to banish entirely from medical nomenclature the terms puerperal and child-bed fever, and to substitute in their place that of uterine inflammation, or inflammation of the uterus and its appendages in puerperal women. The confusion and error which have hitherto prevailed on this subject have recently been aggravated by a futile attempt to include under the term 'puerperal fevers,' not merely the disease usually known by the name puerperal fever, but puerperal intestinal irritation, which is a totally distinct affection; hysteralgia, or what has incorrectly been called false peritonitis, and milk fever. 'If, under the head of *puerperal fevers,*' says the author of this new and unscientific arrangement, 'we are to include all the fevers to which lying-in women are liable, and that are peculiar to the puerperal state, we must not pass over what is commonly called *milk fever.*'

"Until a recent period, the pathological anatomy of the uterine organs in puerperal women had not received the attention which its importance demanded. In the histories of the different epidemic fevers which have prevailed among lying-in women since the middle of the 17th century, the symptoms and the morbid appearances, though imperfectly described, nevertheless strongly confirm the conclusion that the whole phenomena, local and general of these fevers, are to be referred to inflammation of the uterine organs, and that the symptoms vary according as the superficial or the deeper-seated structures are affected. The winter of 1746 at Paris was most destructive to puerperal women, and they died between the fifth and seventeenth day after their confinement. The epidemic attacked the indigent, but much less frequently and severely those women who were delivered at their own habitations than in the Hôtel-Dieu. Of twenty women in child-bed, affected with the disease in February of that year, in the Hôtel-Dieu, scarcely one recovered."

In a number of cases the author throws his facts into a tabular form, by which the reader is enabled at a glance to see the whole bearing of a subject. Thus, he gives a table of 127 cases of *difficult labour*, in which delivery was effected by the operation of craniotomy. The causes of difficulty, the time in labour, and the result in each case are given in little space and the most convenient form. Again, he presents a tabular view of 71 cases of shoulder and arm presentations; and a table, also, of 38 cases of *uterine hemorrhage from placental presentations;* one of 39 cases of severe *uterine hemorrhage, occasioned by detachment of the placenta from the superior part of the uterus;* one of 54 cases of *puerperal convulsions;* and one of 160 cases, covering nearly thirty pages, of *severe inflammation of the uterus and its appendages.* In the latter, not only are the symptoms, the result and morbid appearances in each case stated, but a brief summary of the treatment is also given. This is a compendious and convenient mode of exhibiting such histories, and the value of Dr. Lee's work is very much enhanced by his tables.

We have experienced no little trouble in looking through this work to find passages of moderate length, which could be detached and do justice to the author, and are pretty well persuaded, at length, that to judge fairly of the interest of these lectures at least an entire one must be read. We will, however, venture upon one more extract. It is from the lecture on diseases of the brain in puerperal women, and proceeds thus:

"Miliary fever, intestinal and remittent fever, ephemeral fever or weed, pneumonia, bronchocele, strangury, paralysis, diarrhœa, tympanitis, and spasmodic and nervous disorders, have all been enumerated by systematic writers as peculiar to puerperal women; but as I have no observation of the slightest importance to make respecting these, I shall conclude this department of the course—the pathology of the puerperal state—with a few very general remarks on diseases of the brain and nervous system, and of the nipples and mammæ. From sympathy with the great uterine system of nerves after delivery, there is often a predisposition manifested to disease in the brain, and violent cerebral derangement is produced by causes which, at other times, would have had no effect. In the 5th volume of the Transactions of the London College of Physicians, there is a paper, by Dr. J. Clarke, on the Effects of Certain Articles of Food, especially of Oysters, after Child-birth, in which are related fatal cases which followed the use of oysters in the puerperal state; and the same effect has been produced by other articles of diet. Fourteen days after delivery, a lady was attacked with giddiness, then with violent pain of the head, neither of which yielded to the common remedies. The symptoms increased rapidly; she became senseless and delirious, and convulsions and death followed. Towards the close of the disease it was ascertained that she had eaten oysters on the day preceding the attack. A young, healthy plethoric woman, three weeks after a natural labour, was attacked with pain of the head. A day before she had eaten five or ten oysters, which had been dressed by boiling them in the liquor contained in the shells. Venesection, emetics, and purgatives, were employed, and the symptoms were mitigated for a time; but convulsions came on, and compression of the brain, and she died in no long time. Dr. J. Clarke has also related the case of another young healthy woman, who began a few days after delivery to complain of head-ache, and a sense of internal fulness in the head. These symptoms not having yieled to bleeding, purging, and low diet, a state of coma came on, and she died in sixteen hours. On the day preceding the attack she had eaten twelve raw oysters. On the third day after a natural labour, a woman, who was recovering in the most favorable manner, was suddenly attacked with a fit of cold shivering, followed by violent head-ache, throbbing of the temporal arteries; intolerance of light and sound, and other symptoms which indicated the existence of a dangerous affection of the brain. The pulse was 130; skin hot; tongue loaded. The abdomen was soft and puffy, and not affected by pressure. I ascertained that the attack was produced by the patient having eaten a quantity of ham twelve hours before. The symptoms were relieved by calomel and purgatives. Another woman on the eighth day after a natural labour, was suddenly attacked with insensibility and convulsions. Twenty ounces of blood were taken away by cupping from the temples, and repeated doses of active cathartics were administered, and she recovered. A few hours before the attack she partook rather heartily of roasted chicken and salted tongue, with a friend who came to visit her.

"Inflammation of the brain and its membranes, in puerperal women, is not a disease of frequent occurrence. The symptoms are those of common phrenitis, not of insanity. There is head-ache, flushing of the face, throbbing of the arteries, intolerance of light and sound, delirium. General and local bloodletting, and active purgatives, should be had recourse to as soon as possible, and all the other remedies usually employed in inflammation of the brain: the patient should be kept in a dark room, and ice in a bladder applied over the shaven scalp. Puerperal mania is a much more common disease than inflammation of the brain, and the symptoms and treatment do not essentially differ from mania as it occurs in women who have not been pregnant, or even in the male sex. Without knowing the history of the patient, it would be impossible in any case to ascertain, from the phenomena alone, whether the disease had followed delivery. In puerperal women it assumes all the varied forms of ordinary insanity, occurring sometimes with symptoms of deep depression or melancholy, and at other times with symptoms of great excitement—talking incessantly and vehemently, pouring forth a torrent of words, and running from one subject to another without the least order in their ideas. In some cases the disease first manifests itself by want of sleep and restless nights, and a peculiar irritability of temper, the temper being ruffled at the merest trifles. Often it is first marked by some unreasonable dislike to the nurse, and she is accused unjustly of carelessness and inattention, or the husband is charged with infidelity, or some other crime. In some cases the aberration of mind is preceded by head-ache and other symptoms of cerebral irritation, quick pulse, and hot skin; while in others there is little derangement of the bodily functions, the circulation being almost natural, the skin cool, and the digestive organs very slightly disordered. Sometimes puerperal insanity is preceded by an attack of violent uterine irritation; there is pain of the hypogastrium, increased by pressure, and some other symptoms of uterine inflammation, which have speedily disappeared on the supervention of the mental disorder. In the greater number of cases puerperal mania occurs after natural labours, and has not been preceded by peculiar derangement of the digestive organs during pregnancy, or uterine hemorrhage during labour.

"There is an hereditary predisposition to the disease in a considerable number of those who become affected, and some have suffered from it repeatedly in the puerperal state. The disease is attended with much greater danger when it occurs soon after delivery, with symptoms of violent excitement of the brain, than when it commences several months after, during suckling, in the form of melancholy madness. Dr. Burrows published a table of 57 cases, of which 10 died. Of 92 patients at the Saltpetrière, 6 died, or one in 15. Of these 92 cases recorded by M. Esquirol, 16 occurred from the first to the fourth day after delivery; 21 from the fifth to the fifteenth day; 17 from the sixteenth to the sixtieth day; 19 from the sixtieth day to the twelfth month of suckling; and in 19 cases it appeared after voluntary or forced weaning. M. Esquirol infers that puerperal mania occurs more frequently as the consequence of delivery than of nursing, and that the more distant the time at which it occurs from the period of delivery, the less it is to be feared. Of these 92 cases, 8 were idiotical; 35 melancholic; and 49 maniacal. The ages of these numbers were as follows:—Twenty-two from 20 to 35 years of age; forty-one from 25 to 30; and twelve were upwards of 40; therefore, neither delivery nor suckling, according to this statement, modify derangement as far as age is concerned, for in this disease, produced by other circumstances, it most frequently occurs between the ages of 30 and 35. 63 of the cases related by M. Esquirol were married women; 29 single. 56 out of the 92 were entirely restored to health; 38 of these recovered in the first six months. The appearances on dissection, it is stated, threw no light on the nature of the disease. These 92 cases of puerperal mania were treated by mild purgatives, frequently repeated, by blisters to the nape of the neck, and limbs, enemata, and baths; bleeding was seldom indicated. In the treatment of puerperal mania the greatest attention should be paid to the state of the brain and of the digestive organs. If the countenance is flushed, the carotid and temporal arteries throbbing violently, the head should be shaved, and blood drawn by leeches from the temples. When there is strong general excitement of the circulation, combined with these symptoms of local determination to the brain, blood should be drawn from the arm in quantity proportioned to the severity of the symptoms. In ordinary mania, decided benefit is sometimes produced by moderate general depletion, and after this great advantage follows the application of ice, in a bladder, to the scalp, or a cold evaporating lotion. Light and noise should be carefully excluded, and a nurse accustomed to the charge of insane persons should have the care of the person. It must be a mild, but vigilant and firm control, which is exercised, and especial care taken that patients, who are sometimes violent and vindictive, inflict no injury upon themselves or those around them. The windows should be secured, and all sharp and cutting instruments should be put out of their way. Women are not unfrequently, at the commencement of the disease at least, fully aware of the disordered state of their minds; they feel that they have not their wonted power over trains of thought passing through their minds, and their ideas succeed one another with extraordinary rapidity, and in a manner different from what they did in a state of health. The condition of the mind during sleep or dreaming is also peculiar. Often, when the disease is forming, the ideas have all a relation to the process of delivery. A lady now afflicted with puerperal mania was delivered unexpectedly at the end of the sixth month of pregnancy, when sitting up—the child literally dropped from her, and had nearly fallen upon the floor; she cannot now, though several months have elapsed, be persuaded that the same accident is not about to take place, and keeps her hands nearly all day long in a position to prevent it. The mother of this lady had one or more attacks of puerperal mania, and the disease was obviously hereditary. A disordered state of the digestive organs is not unfrequently a concomitant of puerperal insanity, and in a few cases this has appeared to be the exciting cause of the complaint, for relief has almost immediately followed the action of brisk cathartics. Whether we view deranged states of the digestive organs as causes or consequences of puerperal mania, these should be effectually removed by repeated doses of calomel and purgatives, and the condition of the alvine evacuations frequently ascertained. Bleeding, general and local, is not required in the greater number of cases of puerperal mania, and the frequent and long-continued use of active cathartics is injurious, especially when the disease is accompanied with symptoms of general debility and exhaustion. More benefit is then derived from narcotics, especially acetate of morphia, nourishing diet, and gentle exercise in the open air. Benefit has also been obtained, in some cases, after the acute stage of the disease has passed away, and convalescence has commenced, by allowing the patient occasionally to communicate with her relatives, from whom she had been separated."

The lecture on the history and demonstration of the *nerves of the uterus*, is one of very great interest. We make room for a few extracts from it. The author states that up to the "beginning of the year 1838 there were no preparations in Great Britain showing the nerves of the human uterus dissected, either in the unimpregnated or gravid state, or in the uterus of any of the lower animals." And he adds that Sir A. Cooper then maintained that it was impossible for the nerves of the uterus, or the nerves of any other organ, to increase under any circumstances. The correctness of this opinion seems to have been disproved by our author, who goes on with the following interesting history:

"On the 8th of April, 1838, while dissecting this gravid uterus upon the table of seven months, all the veins of which were injected, I accidentally observed what appeared to be the trunk of a large nerve proceeding upward from the cervix to the body of the uterus along with the right uterine vein, and sending off branches in its course to the posterior surface of the uterus, some of which accompanied the ramifications of the veins, and others were inserted into the peritoneum; this broad band, resembling a plexus of nerves, extended across the posterior surface of the uterus, and covered the nerve midway between the fundus and the cervix. On the left side the same appearances were seen, and several branches of the nerves accompanying the uterine vein were distinctly continuous with branches of the great plexus covering the body of the uterus. As all the blood-vessels and nerves had been cut away close to the neck of the uterus, it was impossible to trace these nerves on the body of the uterus back to the hypogastric and sacral nerves, and demonstrate their continuity with these; but I had no doubt that they were the uterine nerves enlarged by pregnancy. Several eminent anatomists to whom I showed the preparation thought that I had been misled by appearances, and that they were absorbent vessels accompanying the veins and tendinous fibres spread across the posterior surface of the uterus.

"On the 18th December, 1838, a woman in the sixth month of pregnancy died in St. George's Hospital, a few hours after the fœtus and its appendages had been expelled. The uterus was removed with all its blood-vessels and nerves remaining connected with it: the whole of the great sympathetic was taken out, and the nerves were carefully traced from their origins to the different parts of the uterus, while the preparation was covered with alcohol. About eight months were spent in treating the spermatic, hypogastric, and sacral nerves to the uterus, and their distribution over the organ. This could never have been accomplished, I believe, had the dissection been made in air or water."

The splendid work of Professor Moreau differs widely in its character from the modest, unpretending volume we have been considering. The lectures of Dr. Lee are presented to the public in the plainest and least costly dress. It has a great number of engravings, but they are executed in an un-unexpensive style. The aim of the publishers was to give to the profession one of the very best systems of midwifery in the language for the least money—to place a volume of unquestionable excellence within the reach of every medical man in the country. They have been successful, and while they have avoided all unnecessary ornament, they have employed all the illustration that is necessary to exhibit to the eye of the student what is explained in the text. The other volume is one upon which no expence has been spared. It was intended to be the most splendid obstetrical work ever published in America, and we suppose it will be admitted to be without a competitor—certainly nothing equal to it has come under our observation. It is upon the same scale with Dr. Goddard's beautiful volume on the Teeth, and the superb work of Professor Pancoast on Operative Surgery.

We shall not undertake an analytical review of this volume. We have placed it and the work of Dr. Lee side by side, appearing to us, as they do, to be fit associates, and to constitute as complete an obstetrical library as any two volumes in the language could make. And if they, together, will not make an obstetrician, then we are free to confess our conviction, that one cannot be made by books alone. In these eleborate works is contained the experience of two eminent practitioners long versed in the obstetric art. The amplest instructions are given for all the emergencies that can arise; and the whole is illustrated by engravings that render the subjects as intelligible as art can make them. Everything connected with delivery is shown by the plates in the latter work of a size, and with a faithfulness to nature, which nothing but nature could surpass. Such a work is invaluable to the young accoucheur. If he wishes to understand how the forceps are applied—there it is in the book, nearly as large as life. He wishes to study the process of turning, in cases of arm presentations—he turns to the volume, and there is a plate showing the arm descended, and another exhibiting the hand of the surgeon in the act of turning—all *in situ*—as happily translated by an obstetrical teacher in one of our medical schools, "*in sight.*" Well do we remember the fear and trembling with which we went to the first case of labour, in which we expected the use of forceps would be necessary. We had never witnessed the application of the instrument, and had only a theoretical knowledge of the mode of its application. What would we not have given for the inspection of this volume for an hour! In futute the young practitioner may be saved all such embarrassment. In the works which stand at the head of this article, guides are provided for him which leave nothing to be desirad. If any reader suspects this to be an overstrained eulogy, we beg him to avail himself of the earliest opportunity to examine the works for himself, and if then he dismiss not his suspicions, we shall be ready to abdicate the editorial tripod.

In his introduction, Professor Moreau lays down the following order, as the one he means to pursue:

"The first part of the work will be devoted to the explanation of the organs which concur, in any manner, in the accomplishment of the various functions of reproduction, pertaining to the domain of women alone. These organs will be studied,

"1st. In their physiological state, and in a state of rest.

"2d. In their physiological state, and during the performance of their functions.

"3d. In the anomalies which they present, whenever only, however, these anomalies can modify, oppose, or prevent the execution of the functions peculiar to these organs.

"We shall study in the same order the products of these functions, that is, the various parts which enter into the composition of the human ovum.

"When, on this hand, we know the organs, and on the other their products, we shall examine their functions collectively. The second part will include the study of these functions.

"We shall commence by indicating the conditions which denote the aptitude for their occomplishment, which will furnish us an opportunity of writing the history of the catamenia and its varieties. The subject of generation is left to treatises on physiology: we shall say a word or two on conception, and begin immediately the history of pregnancy. We shall establish its divisions, explain its regular course, the signs which characterize it, the means of appreciating the correct value of each one of them; and shall then treat of its duration and termination.

"The third part will be particularly confined to the study of the various terminations of pregnancy, that is, to labour. We shall establish a distinction between labours, and indicate the numerous varieties they may offer, either as terminating by the efforts of nature, alone, or as requiring the interposition of art.

"In the fourth part we shall examine the anomalies of pregnancy in its progress, development, product, location, and termination: when we shall treat of abortion, moles, or false conception, extra-uterine pregnancies, and ruptures of the uterus.

"In a fifth part we shall treat of the natural phenomena which supervene in the woman after delivery, and in the child at birth, then the peculiar cares required by both. To this will be limited the task we have undertaken."

Such is an outline of this great work, from which perhaps the reader will be able to form a rather more satisfactory idea of its proportions and finish, than could be formed of the edifice from the single brick, which the proprietor carried about with him as a specimen. If we could transfer to our pages one of the superb engravings by which the text is illustrated, the case would be very different. That would be the highest recommendation that could be given of the work. Truly may it be said in reference to the task of both author and publishers, 'give them of the fruit of their hands, and let their works praise them in the gates.' The enterprise of the publish is worthy of praise, and we trust will be met by the profession in a liberal spirit. Such works as those of which Moreau's Midwifery is one of a series, cannot be published except at great expence, and as they are calculated to diffuse the most accurate information on subjects which can only be made clear by such illustrations, it is the interest of the profession to foster the enterprise to which we are indebted for them. Y.

